# COMP Report: An updated algorithm to estimate medical physics staffing levels for radiation oncology

**DOI:** 10.1002/acm2.13364

**Published:** 2021-07-28

**Authors:** Kyle E. Malkoske, Katharina E. Sixel, Robert Hunter, Jerry J. Battista

**Affiliations:** ^1^ Simcoe Muskoka Regional Cancer Program Royal Victoria Regional Health Centre Barrie ON Canada; ^2^ Department of Medical Physics Durham Regional Cancer Centre Lakeridge Health Oshawa ON Canada; ^3^ Department of Radiation Oncology University of Toronto Toronto ON Canada; ^4^ Department of Medical Physics Juravinski Cancer Centre Hamilton Health Sciences Hamilton ON Canada; ^5^ School of Interdisciplinary Science McMaster University Hamilton ON Canada; ^6^ Departments of Oncology and Medical Biophysics Western University London ON Canada

**Keywords:** medical physics, radiation oncology, staffing

## Abstract

**Purpose:**

Medical physics staffing models require periodic review due to the rapid evolution of technology and clinical techniques in radiation oncology. We present an update to a grid‐based physics staffing algorithm for radiation oncology (originally published in 2012) that has been widely used in Canada over the last decade.

**Materials and Methods:**

The physics staffing algorithm structure was modified to improve the clarity and consistency of input data. We collected information on clinical procedures, equipment inventory, and teaching activities from 15 radiation treatment centers in the province of Ontario from April 1, 2018, to March 31, 2019. Using these data sets, the algorithm's weighting parameters were adjusted to align the prediction of full‐time equivalent (FTE) personnel with actual staffing levels in Ontario. The algorithm computes FTE estimates for medical physicists, physics assistants, engineering (electrical and mechanical), and information technology (IT) support. The performance of the algorithm was also tested in eight Canadian cancer centers outside of Ontario.

**Results:**

The mean difference between the algorithm and actual staffing for the 23 Canadian cancer centers did not exceed 0.5 FTE for any staffing group. The results were slightly better in Ontario than in other provinces, as expected since the algorithm was optimized using Ontario data. There was a linear correlation between the algorithm predictions and the number of annual‐treated cases for physicists, and physicists plus physics assistants. For other staff categories, the algorithm weighting parameters were not significantly altered, except for a reduction in mechanical engineering staff. Comparison with other published models suggests that the updated algorithm should be considered as a minimum recommended staffing level for the clinical support of radiation oncology programs.

**Conclusions:**

We support the use of grid‐based physics staffing algorithms that account for clinical workload with flexibility to adapt to local conditions with variable academic and research demands.

## 1 INTRODUCTION

Medical physics departments support the systems, infrastructure, and technical procedures necessary to deliver cancer radiation treatment safely and effectively. These activities include but are not limited to: selection, acceptance, commissioning, ongoing maintenance, and quality assurance (QA) of radiation treatment equipment; implementation of advanced radiation treatment techniques and clinical protocols; providing scientific and technical advice on radiation treatment planning; clinical training of students and staff in the field of medical physics and its affiliated radiation specialties; licensing and radiation safety. In addition to these essential clinical services, many departments participate in research and development initiatives to help further advance clinical practice.

Radiation oncology is a rapidly evolving and technology‐driven field of medicine. It is, therefore, important to reassess staffing algorithms at regular intervals and consider the adjustment of human resources plans. Over the last decade, several methodologies have been used to estimate medical physics human resources requirements in radiation oncology.^1^, ^2^, ^3^, ^4^, ^5^, ^6^, ^7^ These methods range from simple scaling factors normalized per number of annually treated patients, to detailed models based on an inventory of clinical workload, equipment, academic activity, and administrative or regulatory oversight. Battista et al. reported a staffing algorithm that was developed in Ontario and extensively validated across Canadian cancer centers (herein this algorithm is referred to as “Ontario‐2012”).^1^, ^2^, ^8^, ^9^ The authors recommended a grid‐based algorithm when evaluating staffing levels for individual physics departments, as simpler algorithms based only on caseload predicted staffing levels less accurately. In the development of the original algorithm, the authors inferred a “rule‐of‐thumb” allocating one full‐time physicist (FTE) per 260 annual radiotherapy cases, to *estimate* staffing levels at a broad population level only (e.g., provincial projections). This ratio was consistent with a subsequent report from the United States in 2015, where the median number of patients per qualified medical physicist was 250.^10^

A survey of physics departments in Ontario cancer centers was performed in 2018, using the Ontario‐2012 algorithm. The survey results indicated a growing discrepancy between the algorithm predictions and actual staffing levels in the province. We suspected this disparity was attributable to ambiguity in the 2012 algorithm's input parameters (e.g., case complexity, major versus minor equipment), and technological progression that had reduced the amount of medical physicist resources required to support intensity‐modulated radiotherapy (IMRT). Adding to the motivation to revisit the original algorithm, the Ontario provincial government is currently revising its funding model for radiation therapy, and evaluating methods to account for physics workload, particularly those components not linked directly to patient care such as equipment support. Given these motivating factors, the purpose of this work was to review, reorganize, and update the parameters used in the Ontario‐2012 algorithm to better align the algorithm with current clinical practice in established cancer centers.

## 2 MATERIALS AND METHODS

The staffing algorithm estimates the required number of FTE positions based on a nominal 37.5‐h work week (i.e., 1950 h per year). The algorithm computes FTE estimates for medical physicists, physics assistants (or associates), engineering (electrical and mechanical), and information technology (IT) specialists (Table 1). In jurisdictions where roles may be blended, the algorithm's FTE components can be re‐grouped. For example, physics assistants are typically not employed as a separate category in the province of Quebec, and therefore the algorithm's physics assistant FTEs may be added to the physicist FTEs. The calculation of dosimetrist (or treatment planner) FTE was removed from this iteration of the algorithm, because these staff members are more commonly included in the radiation therapist (i.e., medical radiation technologist) staffing model utilized in Ontario.^16^ However, the workload associated with technical supervision of dosimetrists remains within the scope of practice of medical physicists in Ontario, and is accounted for in this algorithm.

**TABLE 1:** Full‐time equivalent (FTE) positions included in the staffing algorithm

Position	Responsibilities	Typical education/certification
Medical Physicist	Systems, technology, and scientific oversight of the radiation treatment program. Research, development, and education activities.^11^, ^12^	PhD or MSc Certified by Canadian College of Physicists in Medicine, American Board of Radiology, American Board of Medical Physics, or equivalent.
Physics Assistant	Quality control measurements and other specific tasks under the supervision of medical physicists.^13^, ^14^, ^15^	MSc or BSc
Engineering ‐ Electrical	Service/repair of radiation treatment equipment.	Electronics Engineering Technology (diploma)
Engineering ‐ Mechanical	Mechanical repairs of radiation treatment equipment and patient accessories. Fabrication of various QA devices.	Machinist/Mechanical Technician (diploma)
Information Technology	Software/network support of radiation treatment and QA systems.	Network Administration (diploma)

The staffing algorithm was reorganized into five major components as described in Table 2. Some key assumptions about the activities that are included in each component are also listed to guide the user. Naturally, variations in medical physics duties exist, depending on local needs, and funding. The algorithm tool not only provides default Ontario‐based FTE weightings, but also customization options for centers to adjust the algorithm weights to better reflect unique aspects of their clinical and, if applicable, academic workload. The “weight” is the fraction of an FTE required to perform the tasks associated with the listed duty. Instead of performing a detailed study of the time required to perform individual tasks,^10^ we used Ontario staffing levels between April 1, 2018, and March 31, 2019, as a reference dataset for setting the algorithm's default weights. The staffing levels at this time were felt to be reasonable, as provincial waiting times were within targets^17^ and there were no reported radiation treatment incidents attributable to limited physics staffing resources. All staffing estimates using the updated version of the algorithm with (Ontario‐optimized) default weights are herein referred to as “Ontario‐2021.”

**TABLE 2:** Components of the Ontario‐2021 physics staffing algorithm

Staffing component	Inclusions
Clinical Procedures	Clinical support for individual patients including clinical consultations (pre‐, or while on‐treatment), treatment plan/chart review, patient‐specific QA, *in*‐*vivo* dosimetry.
Clinical Equipment	Equipment selection, installation, acceptance, and commissioning. Routine maintenance and QA. Licensing and radiation safety. Training of clinical staff on equipment usage.
Core Services	Protocol/treatment technique development, implementation, and maintenance. Radiation incident investigation and learning. Quality assurance program oversight.
Education and Training	Training of future staff including residents, students, and classroom teaching in medical physics and associated professions such as radiation oncology and radiation therapy technology.
Administration	Supervision of departmental staff, vacations, conferences, site visits, continuing education.

### 2.1 Updates to physics staffing algorithm

The updated Ontario‐2021 weighting parameters are summarized in Tables 3 and 4. For example, a medical physicist is assigned a baseline weight of 0.5 FTE per 1000 treated cases (any modality), with an additional weight of 0.1 FTE per 100 complex cases, 0.25 FTE per 100 highly specialized cases, and 0.2 FTE per 100 brachytherapy fractions. For clinical equipment, the normalizing factor is the number of units or systems, for example, 0.2 medical physicists per standard Linac. Most software elements are considered within a networked system in this updated version of the algorithm (distinguished only by vendor), as the number of workstations is less relevant in thin‐client or cloud‐based installations of clinical software. A further explanation of the algorithm subsections follows.

**TABLE 3:** Default full‐time equivalent (FTE) weighting for clinical procedures and clinical equipment components of the Ontario‐2021 staffing algorithm

Item^a^	Weight normalization per	FTE Weighting				
		Medical physicist	Physics assistant	Engineering electrical	Engineering mechanical	IT support
**Clinical procedures**						
Radiation‐treated cases per year (all modalities)	1000 cases	0.50	0.20	0.10	0.05	0.10
Complex cases (default 25% of annual cases)	100 cases	0.10	0.05	0.02	0.01	0.02
Highly specialized cases (default 1% of annual cases)	100 cases	0.25	0.12	0.05	0.02	0.05
Brachytherapy fractions per year	100 fractions	0.20	0.05	0.01	0.01	0.01
**Clinical equipment**						
*Megavoltage (MV) treatment units*						
Linacs (gantry or robotic), Gamma Knife units	unit	0.20	0.20	0.25	0.05	0.03
MR Linacs	unit	0.40	0.40	0.50	0.10	0.06
Proton Accelerators	unit	2.00	1.00	0.50	0.10	0.06
*Major equipment*						
TPS (external beam, brachytherapy, etc.)	system^b^	0.10	0.05	0.00	0.00	0.03
ROIS	system^b^	0.10	0.05	0.00	0.00	0.20
Simulators (4DCT, MR or PET‐CT), HDR units	unit	0.10	0.05	0.15	0.03	0.03
*Minor equipment*						
Secondary dose calculation software	system^b^	0.05	0.03	0.00	0.00	0.03
SRS collimator (cone) sets, Cobalt−60 units	unit	0.05	0.03	0.10	0.03	0.00
LDR unit/seed implant program, orthovoltage, ultrasound unit, x‐ray (conventional) simulator, x‐ray C‐arm, gating or motion management system	unit or system^b^	0.05	0.03	0.10	0.03	0.03
QA equipment	cancer center	0.20	0.12	0.20	0.06	0.12
Equipment specification, evaluation and procurement	MV, major and minor equipment FTE	2.0%	0.0%	0.0%	0.0%	0.0%
Licensing, and radiation safety officer duties	regulated device or program^c^	0.025	0.00	0.00	0.00	0.00
Training of clinical staff on equipment operations	Linac and TPS	0.02	0.00	0.00	0.00	0.00

^a^ 4DCT, Four‐Dimensional Computed Tomography; HDR, High Dose Rate; LDR, Low Dose Rate; MR, Magnetic Resonance; PET, Positron Emission Tomography; ROIS, Radiation Oncology Information System; SRS, Stereotactic Radiosurgery; TPS, Treatment Planning System.

^b^ A system is characterized by a unique vendor, irrespective of the number of units or workstations.

^c^ Regulated devices or programs include those that require specific national or provincial licensing prior to clinical use (e.g., Linacs, LDR brachytherapy, etc.).

**TABLE 4:** Default full‐time equivalent (FTE) weighting for core services, education/training, and administration components of the Ontario‐2021 staffing algorithm

Item	Weight normalization per	FTE Weighting				
		Medical physicist	Physics assistant	Engineering electrical	Engineering mechanical	IT support
**Core services**						
Clinical protocol development, implementation and maintenance	equipment and procedures FTE	20.0%	0.0%	0.0%	0.0%	0.0%
Radiation incident investigations and quality assurance program oversight	1000 cases	0.04	0.00	0.00	0.00	0.00
**Education and training** ^a^						
Clinical physics residents	resident	0.10	0.025	0.00	0.00	0.00
Radiation therapy and undergraduate students	student	0.02	0.005	0.00	0.00	0.00
Radiation oncology residents	resident	0.01	0.000	0.00	0.00	0.00
Graduate students (MSc, PhD)	student	0.10	0.025	0.00	0.00	0.00
Classroom teaching – university half credit courses	course	0.06	0.000	0.00	0.00	0.00
**Administration**						
Administrative workload applied to department head and supervisors (sum of this row is applied to physicist FTE)	staff member FTE	0.05	0.02	0.02	0.02	0.02
Coverage for statutory holidays, vacation, continuing education	staff member FTE	0.10	0.10	0.10	0.10	0.10

^a^ An additional baseline of 0.1 medical physicist FTE is added to the clinical physics resident, radiation oncology resident, and graduate student rows to account for overall program infrastructure and administration (applicable only if the student count is >0 in the given category).

#### 2.1.1 Clinical procedures

Ontario uses a specific definition of the *treated case* to describe patient caseload. Annual‐treated cases, for a given cancer center, refer to the number of patients that receive treatment for a specific *primary disease*, independent of treatment modality (i.e., external beam or brachytherapy), and ignoring additional metastatic or retreatment courses during the same year. Note that a suitable substitute for treated cases is the number of *distinct* patients treated annually, since the only difference is that the latter excludes patients treated for more than one *primary* disease within the year. Based on the Ontario experience, this only happens rarely in about 1% of patients.

The original algorithm explicitly listed clinical procedures that were deemed *complex*, or *highly specialized* and assigned additional bonus weighting (e.g., IMRT, Stereotactic Radiosurgery (SRS)). While the core principle of this approach has been maintained, the list of procedures has been removed in the new version. Instead, the user specifies a proportion of cases (%) that are deemed to be considered complex or highly specialized. This approach provides better flexibility to account for the ongoing balance between maturing complex procedures which become routine and newly emerging techniques that require more time during the early stages of clinical implementation. Based on the experience in Ontario, default values of 25% and 1% of total cases were found to be reasonable estimates for complex and highly specialized procedures. Using this 25%/1% mix of techniques results in an average of 1.5 h of medical physicist time per case treated with external beam radiotherapy, that is, (0.5/1000 + 0.25 × 0.1/100 + 0.01 × 0.25/100) × 1950 h per annum (Table 3).

It is acknowledged that there is variability in the resources required within the complex and highly specialized categories, as well as between the different types of brachytherapy procedures. The proposed methodology strikes a balance by assuming an average impact on resources within each category.

#### 2.1.2 Clinical equipment

The Ontario‐2012 algorithm categorized equipment as *major* or *minor*, with a list of sample equipment for each category. The updated algorithm requires the user to enter a more specific inventory of clinical equipment, similar to the method used by several other grid‐based models.^4^, ^5^, ^6^, ^7^ The aim was to improve the consistency across centers reporting their equipment inventory. Administrative activities associated with equipment were also included, such as procurement, licensing, and operations training for clinical staff. The default weighting for licensing and radiation safety aspects, typically assigned to the radiation safety officer (RSO), was based on workload information collected by the RSO “Community of Practice” in the province of Ontario.^18^

#### 2.1.3 Core services

This new category was introduced to account for the critical role that medical physics staff plays in safely implementing and maintaining advanced treatment techniques and clinical trials protocols. These components were dispersed throughout the original algorithm and have been re‐grouped in this iteration for more explicit clarity. A default value of 20% of the FTEs allocated to clinical procedures plus equipment support is used to describe this activity for medical physicists. This may be increased, for example, in centers more heavily involved in leading early evaluation and implementation of novel treatment techniques as opposed to adopting well‐established techniques developed by other institutions or industry. Core services also account for medical physicist's involvement in key radiation treatment program quality initiatives, such as quality assurance program oversight and radiation incident reporting and learning.^19^, ^20^

#### 2.1.4 Education and training

This section focuses on the important role of medical physics staff in training future generations of health care professionals working in radiation oncology. This involves classroom teaching, on‐the‐job training, and supervision of medical physics residents, radiation oncology residents, medical physics graduate students, radiation therapy students, and undergraduate term students. The Ontario government provides studentships to undergraduates and supports a large medical physics residency program with up to 24 FTE positions at 15 regional cancer centers across the province, each of which is accredited by the Commission on Accreditation of Medical Physics Education Programs (CAMPEP).^21^

#### 2.1.5 Administration

The workload associated with supervision and human resources requirements of physics staff is estimated for each reporting staff category in the department and applied to the physicist FTE total. Furthermore, an allotment for absences due to vacations, conferences, site visits, and continuing education is included.

### 2.2 Algorithm performance testing

The algorithm was programmed into a Microsoft Excel spreadsheet and is available from the corresponding author upon request. All 15 radiation treatment centers in Ontario completed the spreadsheet, as well as a sample of 8 centers of variable workload selected from other provinces (4 from western Canada, 2 from Quebec, and 2 from Atlantic Canada). The data submitted for treated cases in Ontario were cross‐checked against the Ontario Health (Cancer Care Ontario) clinical registry data repository (i.e., iPort^TM^) and adjusted as necessary.^17^ Centers outside of Ontario used the distinct patient count instead. A sampling period from April 1, 2018, to March 31, 2019, was used in the responses from all centers.

## 3 RESULTS

**FIGURE 1:**
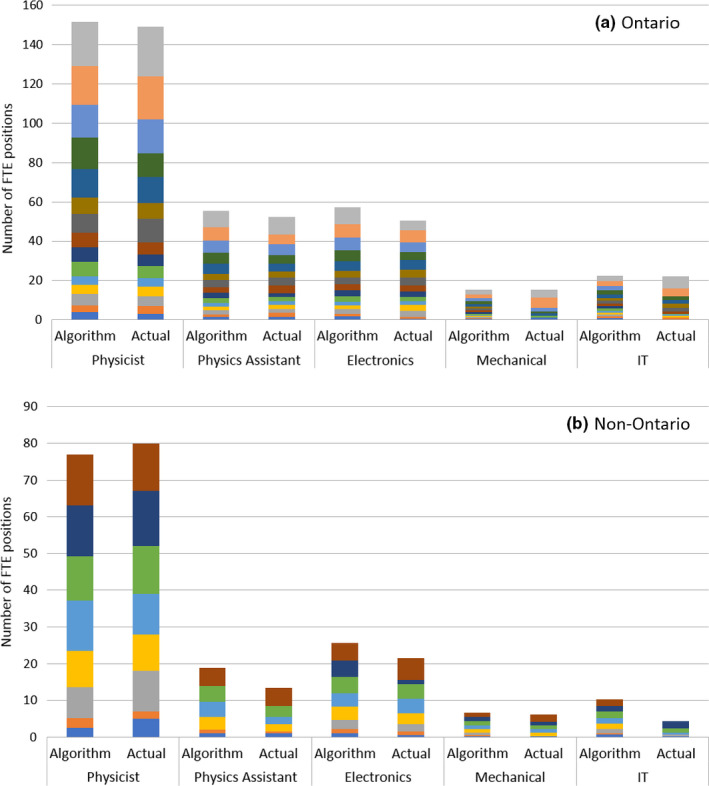
Survey results of the updated staffing algorithm compared with actual staffing levels at 23 Canadian cancer centers (colored blocks). The Ontario centers (a) and non‐Ontario centers (b) are shown separately

**FIGURE 2:**
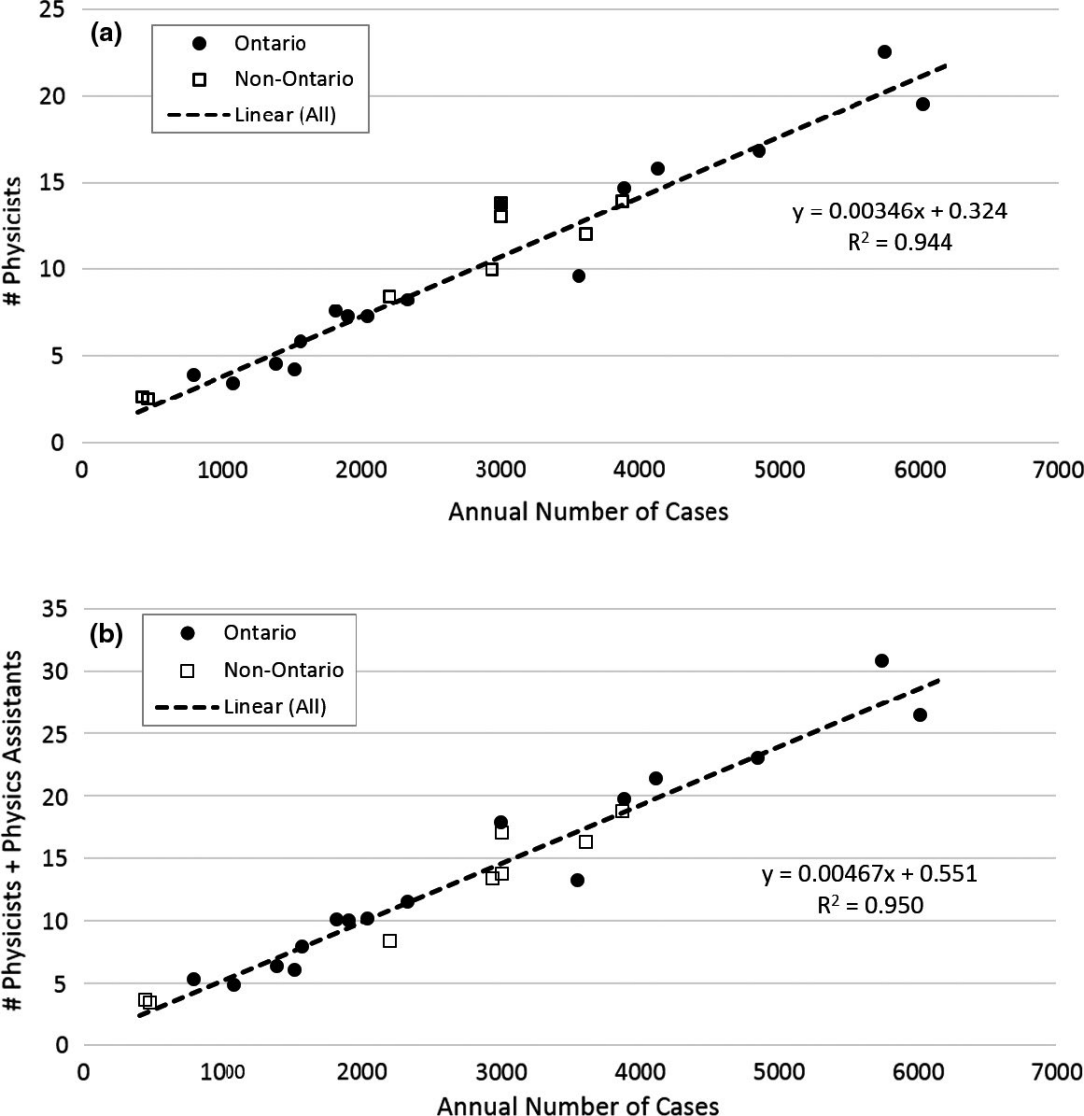
The algorithm full‐time equivalent for physicists (a) and the sum of physicist +physics assistant (b) were linearly correlated with the number of treated cases per year

The staffing estimates using the Ontario‐2021 algorithm are shown in Figure 1 for Ontario and non‐Ontario centers. Each color in the stacked chart corresponds to an individual cancer center, based on the workload and inventories provided in their survey responses. A range of complexity was reported in the initial survey responses, but these were then re‐normalized to standard 25%/1% complexity proportions as described in Section 2.1.1. For centers in the province of Quebec, the physics assistant FTE count was added to the physicist total, for reasons mentioned previously. A summary of the algorithm compliance with actual staffing is shown in Table 5. When applying the default weighting, the mean difference between the algorithm and actual staffing for the 23 Canadian centers surveyed was within 0.5 FTE for all staffing categories. As expected, the mean differences were smaller in Ontario since the algorithm was optimized using Ontario as the reference staffing data set. A linear correlation between the algorithm's FTE for physicists and (physicists plus physics assistants) versus annual caseload was observed as shown in Figure 2. Linear correlations were also observed for electronics engineering, mechanical engineering, and IT support (R^2 ^= 0.93, 0.96, and 0.94, respectively).

**TABLE 5:** Mean difference (±1 s.d.) between the algorithm and actual FTEs for 23 surveyed centers. A positive mean difference indicates that the algorithm (with default weighting) over predicts the actual staffing levels

Region	Algorithm – Actual FTE (mean ±1 s.d.)				
	Physicist	Physics assistant	Engineering		IT support
			electronics	Mechanical	
Ontario (N = 15)	0.2 ± 1.8	0.2 ± 0.8	0.5 ± 1.3	0.0 ± 1.2	0.0 ± 1.3
Non‐Ontario (N = 8)	−0.4 ± 1.8	0.9 ± 0.9	0.5 ± 1.3	0.1 ± 0.4	0.8 ±0.7
Canada (N = 23)	0.0 ± 1.7	0.4 ± 0.9	0.5 ± 1.2	0.0 ± 0.9	0.3 ± 1.2

## 4 DISCUSSION

In 2018, the Ontario‐2012 algorithm predicted a sharp increase in physics staffing levels compared to previous staffing models used in Canada.^9^ This was largely attributable to the rapid proliferation of new technology at the time such as inverse‐planned IMRT and CT image guidance. Changes in required staffing predicted by the current iteration of the staffing algorithm are more restrained as these novel techniques have become streamlined. Mechanical engineering support has been markedly reduced for the reasons discussed below.

In addition to some reorganization of the staffing algorithm structure, this update was essentially a curve‐ fitting exercise to align the algorithm to actual staffing levels in Ontario, based on clinical activity and equipment inventory in the fiscal year 2018/19 (April 1, 2018 to March 31, 2019). The fit parameters (i.e., FTE weights) do not represent a unique solution to this multivariate optimization problem. Furthermore, it is possible that actual staffing at the time may have been budget‐limited and not meeting the clinical need. However, during this same time period, 86.2% of patients in Ontario met the Ontario Health (Cancer Care Ontario) waiting time targets for commencing their radiation treatment.^22^ This slightly exceeds the provincial goal of 85% compliance, and therefore one can rule out the presence of any significant understaffing at the time of the survey. Patient safety was not compromised with this level of staffing; no major radiation incidents due to physics understaffing were reported during this study period.

To evaluate perceived FTE inequities, we also collected information on the *desired* staffing levels at each center during the survey as judged by the head of the medical physics department. For the 23 surveyed centers, the mean difference between the desired and algorithm FTE physicists was 1.0 ± 1.6 (1s.d.). In other words, centers would typically prefer approximately one additional medical physicist over the algorithm prediction with its default weights. This finding confirms that the staffing model was not fit to an anomalously low level of medical physicists in Ontario during the sampling period. Some discrepancy may be due to the nuances of local clinical practices, or extraordinary support of laboratory research and academic programs at local colleges and universities.

Although the algorithm results and actual staffing agreed well on average, the standard deviation of 1.7 FTE for the physicist category across all Canadian centers (Table 5) highlighted some of the limitations of applying the default algorithm weighting. The algorithm offers a custom weighting section where the weights can be adjusted for these special considerations, with local justification. Such customization of weights is expected to yield staffing levels within approximately 10% of the predicted staffing levels using the default FTE weights.

Physics assistants are employed in many Canadian cancer centers. The Ontario‐2021 staffing algorithm suggests a ratio of approximately 0.33 physics assistants per medical physicist. This is a slightly higher physics assistant compliment compared to the recent recommendations of the American Association of Physicists in Medicine, where a maximum of 0.25 physics assistants per medical physicist was proposed.^15^ In regions where physics assistants are not employed, the algorithm's physics assistant FTE should be combined with the physicist FTE total.

The 2018 survey that prompted the update of the Ontario‐2012 algorithm revealed a growing discrepancy between that model's predictions and actual Ontario staffing levels. However, the former ratio of 260 annual‐treated cases per physicist is now only slightly revised upward in the new model to 276 ± 52, based on a simple average of the algorithm‐predicted case ratios in the 23 Canadian cancer centers. This confirms that the main source of the drift was in the misapplication of the 2012 model due to its outdated (or ambiguous) description of input workload parameters. Changes in the definition of case complexity and specification of equipment inventory have had the most significant impact in modernizing the model and improving the accuracy and consistency of its predictions. The updated caseload per FTE ratios for physics assistants, electronics engineering, and IT support was also consistent with the 2012 algorithm. Moreover, the requirements for mechanical engineering support have reduced significantly, with the previous rule of thumb (1200 treated cases per FTE) being completely outside of the range of current algorithm results (mean 2725 treated cases per FTE, with a range of 1552 to 3354). In Ontario, the total mechanical engineering FTE predicted by the updated algorithm matched well with actual reduced staffing at a provincial level. However, center‐specific results showed large variations (Figure 1; Table 5). This is explained by some centralization of mechanical support services to larger centers for the design/fabrication of specialized devices, with 70% of small centers in Ontario (<6 Linacs) reporting that they no longer have any in‐house mechanical engineering (or machinist) staff. The widespread adoption of IMRT and volumetric modulated arc therapy (VMAT) has greatly reduced the need for custom‐made devices, while the introduction of 3D printers has simplified the fabrication of selected custom‐made accessories.^23^ Furthermore, modern Linacs have less requirements for complex mechanical procedures to access components during routine servicing (e.g., hoisting the Linac head). Indeed, many facilities have integrated the positions of mechanical and electronics engineering to a single classification of a service engineer, following the example set by Linac vendors.

In the survey, we did not collect any information on how the maintenance of radiation treatment equipment was locally managed. There is a spectrum of approaches throughout Canada, ranging from maintenance and repairs predominately being performed by local in‐house engineering staff (i.e., no vendor service contract) to full vendor service agreements including upgrades, parts, and labor. The default algorithm weights do not account for this variation explicitly, assuming a shared service model and excluding vendor‐employed FTEs. Nevertheless, the algorithm's electronics engineering FTE agreed reasonably well with actual staffing levels (Figure 1; Table 5). A saturation was noted in the number of actual and desired electronics engineering staff at 6 FTE for centers with ≥10 Linacs, despite algorithm predictions reaching as high as 8.7 FTE for the largest center in Ontario (17 Linacs).

Several organizations have proposed similar staffing models for medical physics. We selected four widely referenced international models from the past decade and compared the results to the Ontario‐2021 algorithm. All these models have a similar structure, with components of physics work separated and explicitly accounted for. The AbtIV data,^10^ commonly referenced in the United States, were excluded from the comparison as it uses a different approach of distributing “non‐procedural time” (equipment support, core services, etc.) across a spectrum of direct patient care procedure billing codes. This makes the method highly dependent on these billing code categories and reporting practices in the United States. In Table 6, we compare the calculations by other models for three representative centers in Ontario, based on the data these centers provided in the survey. For the Ontario results, the sum of physicist and physics assistant is presented, as this distinction is not made in other models. The small center had 1 4DCT simulator, 4 Linacs, and recorded 1500 treated cases annually. The medium center had 2 4DCT simulators, 7 Linacs, HDR brachytherapy, and logged 2300 annual‐treated cases. The large center had 11 Linacs, HDR brachytherapy, multiple simulators (4DCT, MR, and PET‐CT), gamma knife, and orthovoltage, treating almost 5000 distinct patients annually. The Ontario‐2021 model showed the lowest FTE count and the best agreement with current staffing at these selected model centers. The European Commission (EC) model was approximately 10 FTE higher in the medium and large centers, due in part to the large weight put on IMRT cases. There is some ambiguity in the input parameters to these models, which may impact the results presented in Table 6. Improving the clarity of input parameters was one of the motivating factors in re‐organizing and expanding the Ontario‐2021 algorithm. Of the three non‐Ontario models, the IAEA staffing model was closest to the Ontario‐2021 algorithm and actual staffing. Perhaps this is not surprising as there was some past communication with this agency on the topic of staffing algorithms.

**TABLE 6:** Staffing calculations for various published models (with the year of publication in brackets) for three representative centers in Ontario. Physicist and physics assistant full‐time equivalent (FTE) were summed in the Ontario algorithm predictions for consistency. Actual staffing is also shown for reference. Refer to the text for a description of the center size.

Center size	Staffing Model^a^					Actual staffing
	EC (2014)	IAEA (2015)	IPEM (2017)	Ontario−2012	Ontario−2021	
Small	11.0	8.5	8.3	6.9	6.1	7.0
Medium	20.1	14.2	16.3	14.9	11.5	11.0
Large	34.4	25.4	28.0	29.2	23.0	22.5

^a^ EC, European Commission^5^; IAEA, International Atomic Energy Agency^6^; IPEM, Institute of Physics & Engineering in Medicine^7^

While the Ontario‐2021 staffing algorithm showed good agreement with actual staffing at a broad level, it should be noted that in some centers the algorithm's default weights resulted in an underestimation of the number of FTE physicists relative to current staffing levels. In discussing the discrepancies with these centers, some of the common reasons for underprediction by the algorithm included: minimum staffing levels required for clinical coverage (especially in small centers); the lack of including exceptional research workload in the default weights (especially in large academic centers); and local differences in scope of practice, particularly in situations where physicists are more heavily involved in clinical procedures such as routine treatment planning (e.g., stereotactic body radiation treatment (SBRT) or brachytherapy patients), or supervision of daily patient setup for SBRT/SRS. Given these factors, coupled with the smallest FTE in comparison to international models, the Ontario‐2021 algorithm should be considered a *minimum* recommended staffing level for clinical support of radiotherapy programs.

## 5 CONCLUSIONS

The staffing algorithm has been updated to better align with 2018/19 actual staffing levels in Ontario. The algorithm was tested at 23 centers across Canada, yielding slightly better agreement with actual staffing in Ontario than in other provinces. At a broad level, mechanical engineering support was the only staffing category that showed an appreciable drop compared to the previous version of the algorithm. The algorithm was found to produce lower physics FTE (physicist +physics assistant) compared with other models published in the last decade. We recommend the application grid‐based models that account for clinical workload with flexibility to adapt to local conditions, evolving practices, and changing infrastructure.

## CONFLICT OF INTEREST

No conflicts of interest.

## Data Availability

The data that support the findings of this study are available from the corresponding author upon reasonable request.

## References

[1] Battista JJ, Clark BG, Patterson MS, et al. Medical physics staffing levels for radiation oncology: a decade of experience in Ontario, Canada. J Appl Clin Med Phys. 2012;13(1):93‐110. 10.1120/jacmp.v13i1.3704 PMC5716143 22231223

[2] Battista JJ, Clark BG, Patterson MS, et al. Erratum: Medical physics staffing levels for radiation oncology: a decade of experience in Ontario. Canada. J Appl Clin Med Phys. 2012;13(3):146‐147. 30252921 10.1120/jacmp.v13i2.3915 PMC5716419

[3] International Atomic Energy Agency . Planning national radiotherapy services: a practical tool. Human Health Series No. 14. IAEA. Vienna; 2010.

[4] Round WH, Tay YK, Ng KH, et al. AFOMP Policy Statement No. 2: recommended clinical radiation oncology medical physicist staffing levels in AFOMP countries. Aust Phys Eng Sci Med. 2010;33(1):7‐10. 10.1007/s13246-010-0003-y 20237891

[5] European Commission Guidelines on medical physics expert. Radiation Protection No. 174. European Union, Luxembourg; 2014.

[6] International Atomic Energy Agency . Staffing in radiotherapy: an activity based approach. Human Health Reports No. 13. IAEA. Vienna; 2015.

[7] Institute of Physics in Engineering & Medicine . IPEM Policy Statement: recommendations for the provision of a physics service to radiotherapy. IPEM, York; 2017.

[8] Clark BG, Patterson MS, Beaulieu L, et al. Medical physics for radiation treatment: a robust algorithm with trans‐Canada validation. Med Phys. 2011;38(6):3754.

[9] Battista JJ. Medical physics staffing for radiation oncology: predictions and pitfalls. Canadian Org Med Phys Inter newslett. 2019;65(2):11‐14.

[10] American College of Medical Physics . The Abt study of medical physicist work values for radiation oncology physics services: round IV. Bethesda, MD: ACMP; 2015. 10.1016/j.jacr.2005.02.009 17411927

[11] Canadian Organization of Medical Physics . Scope of practice for Canadian certified medical physicists. http://www.comp‐ocpm.ca/_uploads/53jbyv2t9.pdf Accessed April 22, 2021.

[12] Clements JB, Baird TB, de Boer SF, et al. AAPM medical physics practice guideline 10.a.: Scope of practice for clinical medical physics. J Appl Clin Med Phys. 2018;19(6):11‐25. 30338913 10.1002/acm2.12469 PMC6236822

[13] Organization of Medical Physics Associates of Canada. http://www.ompac.ca Accessed April 22, 2021.

[14] American Association of Physicists in Medicine AAPM Professional Policy 29‐A, Medical Physics Assistants: Task delegation and supervision. https://www.aapm.org/org/policies/details.asp?id=361&amp;type=PP Accessed on April 22, 2021.

[15] Siebert JA, Blatnica AP, Clements JB, et al. AAPM medical physics practice guideline 7.a.: supervision of medical physics assistants. J Appl Clin Med Phys. 2020;21(7):11‐15. 10.1002/acm2.12774 PMC7386193 31800151

[16] Smoke M, Ho EP. Staffing model for radiation therapists in Ontario. J Med Radiat Sci. 2015;46:388‐395. 10.1016/j.jmir.2015.08.004 31052119

[17] Cancer Care Ontario, Ontario Health . iPortTM. http://www.cancercareontario.ca/en/data‐research/accessing‐data/iport Accessed December 8, 2020.

[18] Cancer Care Ontario, Ontario Health . Radiation Projects and Programs. http://www.cancercareontario.ca/en/cancer‐care‐ontario/programs/clinical‐services/radiation‐treatment/radiation‐projects, Accessed December 8, 2020.

[19] Huq MS, Fraass BA, Dunscombe PB, et al. The report of Task Group 100 of the AAPM: Application of risk analysis methods to radiation therapy quality management. Med Phys. 2016;43(7):4209‐4262. 27370140 10.1118/1.4947547 PMC4985013

[20] Canadian Partnership for Quality Radiotherapy . Quality Assurance Guidelines for Canadian Radiation Treatment Programs. http://www.cpqr.ca/wp‐content/uploads/2020/03/QRT2019‐12‐04.pdf Accessed December 8, 2020.

[21] Commission on Accreditation of Medical Physics Educational Programs. http://www.campep.org Accessed December 8, 2020.

[22] Cancer Care Ontario, Ontario Health . Radiation Treatment Program Implementation Plan 2019‐2023. http://www.cancercareontario.ca/en/cancer‐care‐ontario/programs/clinical‐services/radiation‐treatment/implementation‐plan Accessed December 8, 2020.

[23] Rooney MK, Rosenberg DM, Braunstein S, et al. Three‐dimensional printing in radiation oncology: a systematic review of the literature. J Appl Clin Med Phys. 2020;21(8):15‐26. 10.1002/acm2.12907 PMC7484837 32459059

